# Operative management of gouty tophi in the region of the olecranon: a case series

**DOI:** 10.1016/j.jseint.2022.02.003

**Published:** 2022-02-23

**Authors:** Joshua D. Kirschenbaum, Ruby G. Patel, Matthew R. Boylan, Mandeep S. Virk

**Affiliations:** Division of Shoulder and Elbow, Department of Orthopedic Surgery, New York University Langone Health, New York, NY, USA

**Keywords:** Olecranon, Olecranon bursa, Tophaceous gout, Elbow, Surgical technique, Operative outcomes

## Abstract

**Background:**

Tophaceous gout affecting the olecranon region can result in local discomfort, skin ulceration, secondary infection, and considerable disability if left untreated. However, there are limited reports of outcomes, including postoperative complications and recurrence after surgical excision of tophaceous gout deposits at the elbow. The aim of this study is to present our surgical technique and minimum one-year outcomes after surgical excision of tophaceous gout involving the elbow.

**Methods:**

A retrospective chart review was performed on all patients from a single surgeon's practice who underwent surgical excision of gouty tophi of the elbow between January 2016 and December 2019. The indications for surgical excision of tophi included failure of medical management, presence of skin ulceration, and/or large gouty tophi. The relevant data pertaining to patient demographics, preoperative findings, intraoperative findings, surgical pathology reports, and short-term postoperative complications were collected through retrospective chart review. Patients were subsequently contacted for a follow-up telehealth visit to assess recurrence of gouty tophi, functional outcomes, and range of motion (ROM) measurements.

**Results:**

Six male patients underwent 7 total procedures (1 bilateral elbow) during the study period. The mean age of the cohort at the time of surgery was 56.0 ± 7.1 years (range: 45.3-63.5). The mean size of the swelling in 2 maximum dimensions was 5.8 × 3.4 cm. There were no intraoperative or immediate postoperative wound complications. There was no recurrence of gouty tophi at a mean follow-up time of 30.8 months (range: 14.0-43.5). Patients reported physiologic ROM (mean flexion-extension arc of 2°-134°) with no pain at final follow-up.

**Conclusion:**

Surgical treatment of tophaceous gout of the elbow is associated with a low risk of wound complication and recurrence.

Gout is the most common cause of inflammatory arthritis in men in the United States, and its prevalence has risen significantly over the last several decades to a rate of 3.9% in 2015-2016, in the United States.^2^^,^^20^ The term ‘tophaceous disease’ in the setting of gout refers specifically to macroscopic depositions of monosodium urate that often form in extra-articular connective tissue structures, including bursae, tendons, and ligaments.^16^ If left untreated, gouty tophi can lead to skin ulceration, infection, and compressive neuropathy, depending on the location and size of a given lesion.^3^^,^^22^ The exact proportion of patients with gout who also display tophaceous deposits is unclear. Some series have reported a less than 10% prevalence while others have reported rates as high as 35%.^6^^,^^13^^,^^14^^,^^18^ Regardless of the true disease burden, given gout's prevalence, the patient population that suffers from tophaceous gout is sizeable.

Although the exact prevalence of gouty tophi affecting the elbow is not known, the olecranon bursa region is one of the most common sites for the accumulation of tophaceous deposits in the upper extremity (Fig. 1).^13^^,^^19^ Tophaceous deposits in the elbow can affect the range of motion, cause a compressive ulnar neuropathy, and lead to ulceration of the overlying skin.^5^^,^^21^^,^^22^ Although urate-lowering therapies such as allopurinol and febuxostat are considered first-line treatments, operative intervention is indicated for large swellings, ulcerated tophi, or tophaceous deposits causing compressive symptoms.^5^^,^^23^ Both open and arthroscopic techniques have been described for surgical treatment of tophaceous gout; open excision is indicated for large tophaceous swellings in the elbow, but wound complications, including flap necrosis, wound infection and dehiscence, have been associated with this approach.^7^^,^^9^^,^^19^

**Figure 1:**
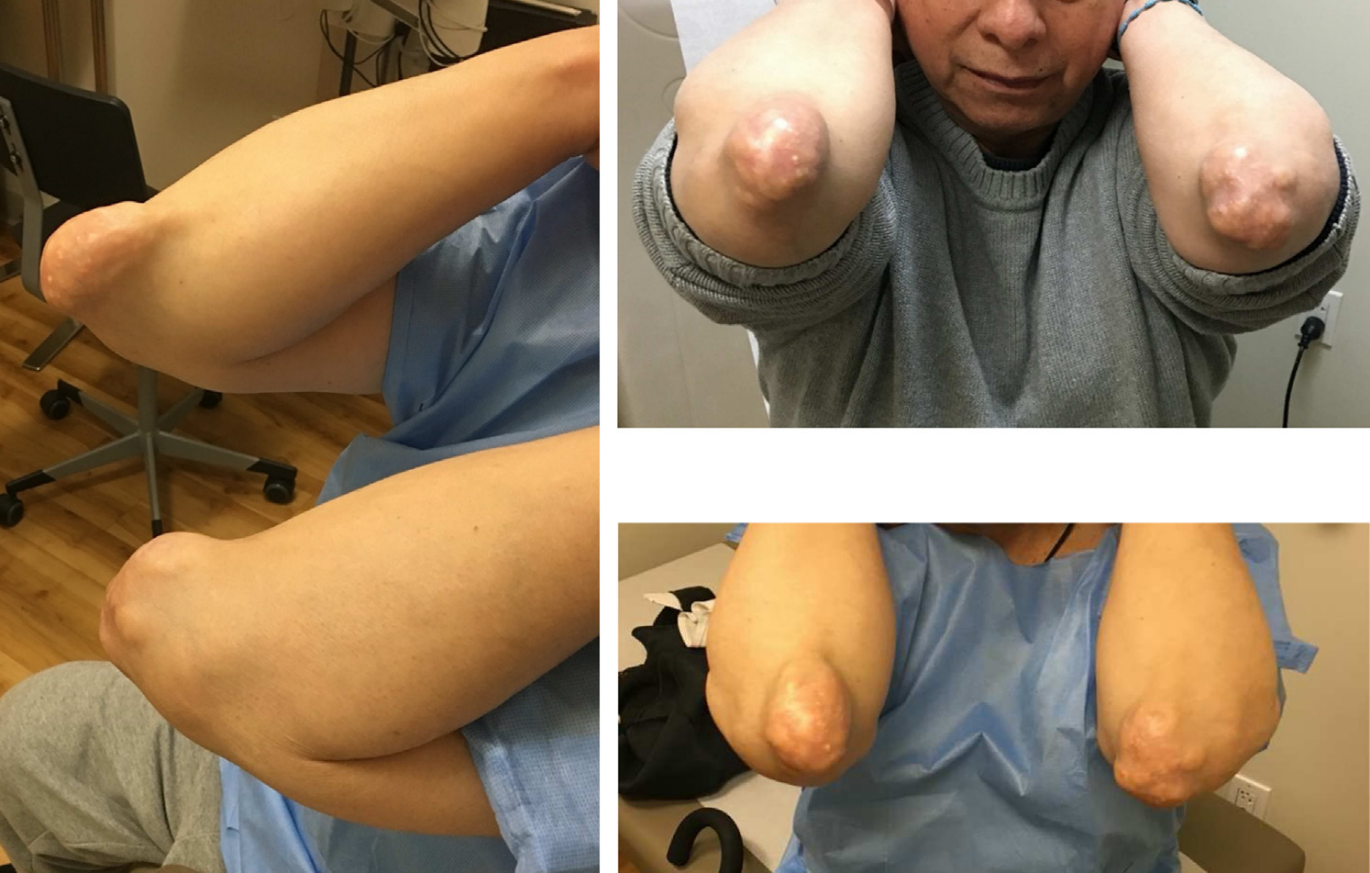
Preoperative clinical picture of a patient with bilateral tophaceous gout deposits affecting the olecranon.

There are few studies that report outcomes after surgical management of tophaceous gout in the elbow, and those published, lack both a thorough description of the surgical technique employed, as well as adequate reported follow-up.^5^^,^^8^^,^^15^ Therefore, the aim of this study is to describe our surgical technique for open excision of gouty tophi in the elbow, as well as to present the corresponding minimum 1-year outcomes for a series of 7 elbows managed with this open excision technique.

## Methods

This case series follows the clinical course of 6 patients (7 elbows) who underwent surgical excision of olecranon gouty tophi in a single surgeon's practice during the study period of 4 years (Jan. 2016-Dec. 2019). Patients of interest were identified from the senior author's surgical database. Exclusion criteria for this study included: (1) active infection of the surgical site, (2) chronic open wound, and (3) presentation for revision surgery. Once eligibility was confirmed, a retrospective electronic chart review (institutional Epic EHR system) was then performed to collect essential demographic data, intraoperative details, complications, and postoperative outcomes. The following information was recorded: sex, BMI, age at the time of procedure, lesion laterality, tophus size, presence of other tophaceous deposits, preoperative serum urate, and medical management of gout. Additionally, patients were contacted for a follow-up visit to assess long-term elbow range of motion (flexion/extension arc), recurrence of tophaceous deposits, incision appearance, and pain. Since this study was performed during the COVID era, the follow-up visits were performed via the telehealth video platform.

### Surgical technique

**Figure 2:**
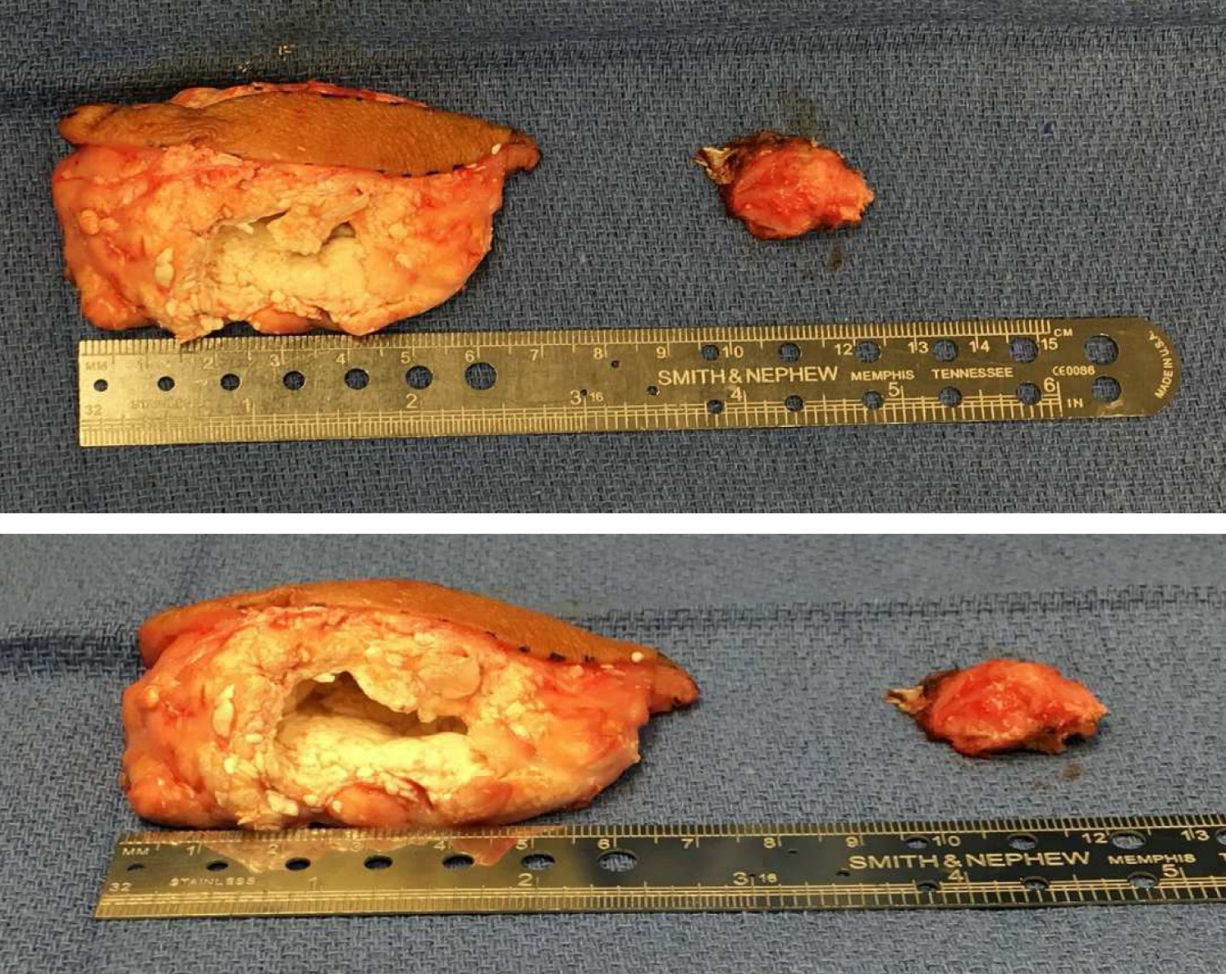
Excised specimen of tophaceous gout of olecranon demonstrating elliptical portion of skin excised along with a large tophus (∼7 cm in maximum dimension).

All surgeries were performed by the senior author under regional anesthesia (single shot brachial plexus block). Local anesthetic infiltration around the incision was strictly avoided before the skin incision or at the end of surgery. A tourniquet was used in all cases. Depending on the size of the swelling, a longitudinal skin incision was made over the olecranon extending beyond the terminal extent of the mass both proximally and distally. In swellings bigger than 5 cm in maximum dimension, an elliptical boat-shaped skin incision was made to excise the redundant part of skin overlying the tophi. All gouty tophi swellings were excised in total; piecemeal dissection should be avoided (Fig. 2). Deeper dissection was performed with a surgical scalpel instead of unipolar electrocautery. It is critical not to perform any blunt subcutaneous dissection in the bulk of swelling. It has been our observation that there exists a natural tissue plane between the gouty mass and underlying deeper tissue (olecranon bone or deep fascia). The best place to find this avascular dissection plane is at the terminal extent of the swelling, either proximally or distally, points at which this tissue plane can be easily developed, and the mass can be excised by continuing the sharp dissection on either side toward the skin incision. The gouty tophus was circumferentially excised using the surgical scalpel, and every effort was made to keep the subcutaneous flaps as thick as possible, even if it meant leaving small chalky white deposits on the flap. Small adherent or satellite tophaceous deposits do not need to be removed from the subcutaneous flaps, as they do not cause recurrence of swelling, especially with continued medical management of gout. While the gouty tophaceous masses are relatively avascular, occasional vascular feeders can be seen at either end of the tophi and should be coagulated with bipolar electrocautery. The excised gouty tophi were submitted for histopathology; an alcohol-based fixative is preferable in these cases as formalin dissolves gout crystals. The hypertrophic olecranon bursal layer, if present, was excised, and bony spurs on the olecranon were débrided with a rongeur and smoothed with a rasp. The triceps tendon insertion was typically not involved, but if tears were present, they were débrided down to vascular, viable tissue. The tourniquet was deflated at this stage of the procedure, and hemostasis was achieved using bipolar electrocautery.

**Figure 3:**
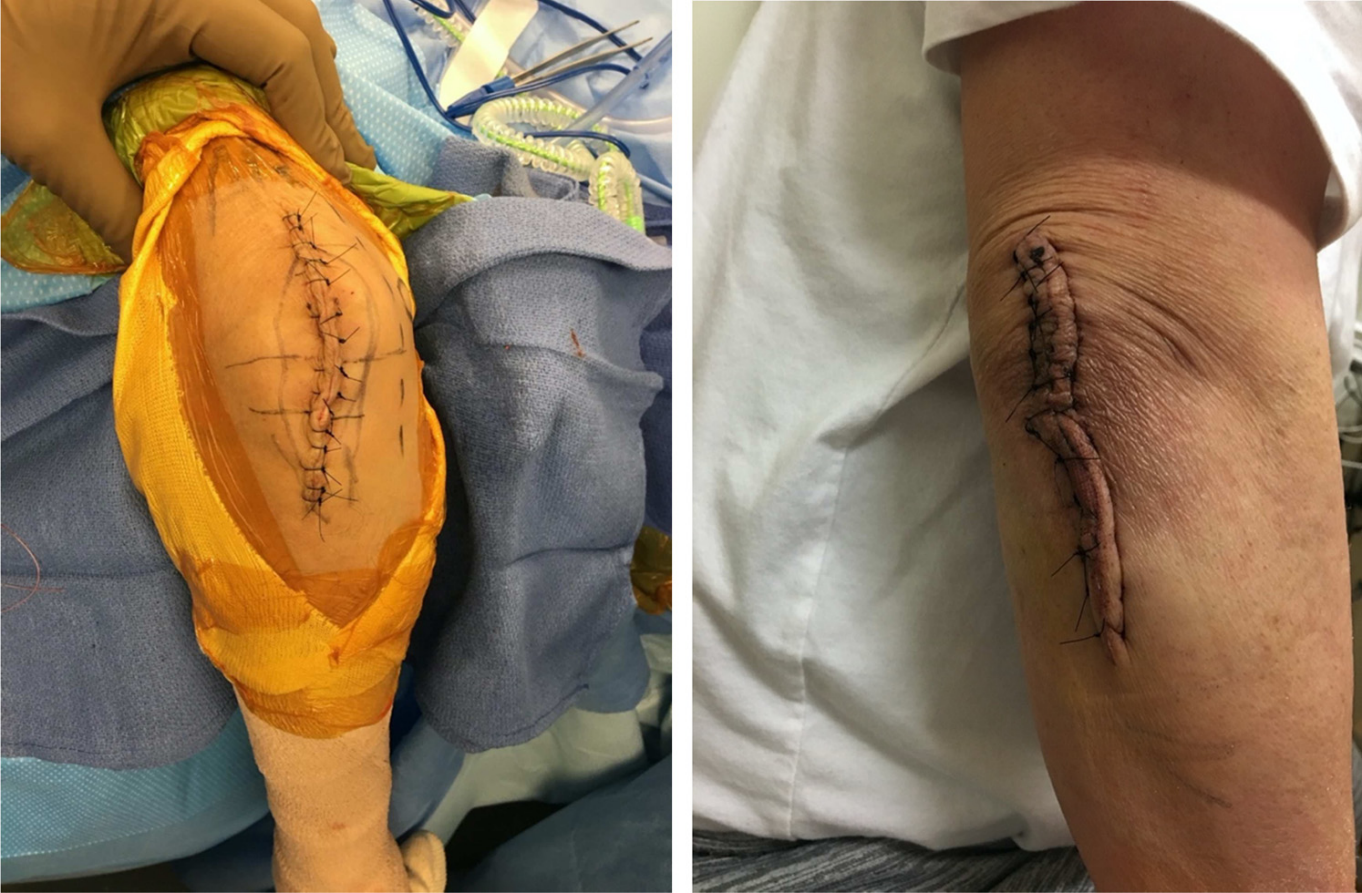
Intraoperative and 2-week clinical photograph demonstrating skin closure and wound healing, respectively.

To reduce wound dead space and the consequent risk of postoperative hematoma, the undersurface of the subcutaneous flaps was tacked to the underlying deep fascia using fine absorbable sutures at the far medial and lateral aspects of the subcutaneous flaps. Typically, no drain was required unless the swelling was very large (>7 cm or so in single maximum dimension), and in those cases, the drain was removed on the first postoperative day. The wound closure was performed with the elbow in 90 degrees of flexion so that the suture line was not under tension when the elbow was flexed during postoperative splint application. Suture material was kept to a minimum in the subcutaneous plane with buried inverted resorbable sutures. Subcuticular sutures were avoided, and the skin incision was closed using #3-0 nylon suture in a horizontal mattress configuration. Care was taken to avoid excessive suture placement, and needle entry point for sutures was kept at least 5-10 mm away from the incisional margins to avoid pressure necrosis along the incision (Fig. 3). All elbows were kept in a posterior elbow splint in flexion for 2 weeks to aid in soft tissue healing and minimize hematoma formation. Sutures were removed at 2 weeks (Fig. 3), and a range of motion exercises was initiated. Direct pressure against the incision was avoided for the next 4 weeks, but no lifting limits were placed on the involved limb.

## Results

Demographic variables from the cohort undergoing surgical removal of olecranon tophi (6 patients, 7 elbows) are listed in Table I. The 6 patients cohort was entirely male, with a mean BMI of 32.8 ± 10.8, a mean age of 56.0 ± 7.1 at the time of the procedure. The mean preoperative serum urate level was 8.8 ± 0.5 (among those who had this lab value recorded, n = 4 elbows), and the mean size of the swellings is shown in Table I. There were 2 right and 5 left elbows operated upon, and all patients except 2 were on a urate-lowering therapy regimen at the time of surgery.

**Table I:** Demographics and preoperative characteristics of gouty tophi.

Patient	Sex	BMI	Age at procedure	Laterality	Maximum tophus size (dimensions in cm)	Other tophaceous deposits	Preop serum uric acid (mg/dL)	Medical management of gout
1 (Left)	Male	31.0	59.4	Left	6.7 × 4.0	Contralateral Elbow Tophus +	9.1	Allopurinol and Colchicine
1 (Right)	Male	31.0	59.2	Right	8.0 × 4.5	Contralateral Elbow tophus +	8.3	Allopurinol and Colchicine
2	Male	27.5	63.5	Right	6.0 × 4.5	None	NA	Febuxostat and Colchicine
3	Male	24.8	48.3	Left	4.2 × 1.9	Contralateral Elbow Tophus +	NA	Allopurinol and Colchicine
4	Male	31.1	53.8	Left	1.5 × 1.0	None reported	8.5	None
5	Male	56.8	45.3	Left	6.7 × 4.0	Contralateral Elbow Tophus +	9.4	Allopurinol and Colchicine
6	Male	27.5	62.8	Left	7.2 × 4.0	None	NA	None
Mean ± SD	-	32.8 ± 10.8	56.0 ± 7.1	-	5.8 × 3.4	-	8.8 ± 0.5	-

*BMI*, body mass index; *NA*, not available.

Intraoperatively, five elbows with swelling >5 cm in a single dimension had an elliptical portion of the skin removed along with the excised gouty tophus (Fig. 2). No intraoperative complications were reported. The primary diagnosis of tophaceous gout was confirmed in all cases upon histopathology; concomitant calcium pyrophosphate crystals were seen in the specimen resected from Patient 4. All incisions were healed at 2 weeks, and no postoperative wound complications were reported in this time frame.

Long-term postoperative outcomes are shown in Table II. Five of the 6 patients, accounting for a total of 5 operative procedures, were successfully contacted and scheduled for a telehealth visit at a mean of 30.8 months postoperatively during this phase of the study. The mean flexion-extension arc of elbow motion was 2°-134°. No patients reported a recurrence of swelling or pain since the surgery, and in all cases, the operative incisions were well healed. No late scar tenderness or breakdown was reported.

**Table II:** Postoperative outcomes (long-term follow-up).

Patient	Time from procedure to final follow-up (months)	Complications by time of most recent follow-up	ROM (F/E)	Recurrence of gouty tophus	Surgical incision at final follow-up	Elbow pain at final follow-up
2	14.0	none	10-130°	No recurrence	Well-healed	0/10
3	42.3	none	0-130°	No recurrence	Well-healed	0/10
4	32.2	none	0-140°	No recurrence	Well-healed	0/10
5	21.8	none	0-140°	No recurrence	Well-healed	0/10
6	43.5	none	0-140°	No recurrence	Well-healed	0/10
Mean/Total	30.76	0/5	2-134°	0/5	-	0/10

Patient 1, who underwent excision of tophaceous gout deposits on the right and left elbows, was unable to be contacted for purposes of long-term follow-up (minimum 1 year for this study) and is, therefore, not included in Table II. At his 2-week follow-up visit for his left elbow (corresponding to 12 weeks postoperatively for his right elbow), incisions were noted to be well healed bilaterally. His postoperative course was notable for an acute gout flare affecting the right wrist in the month after right elbow tophus excision, which resolved with medical management.

*ROM*, range of motion; *F/E*, flexion-extension.

## Discussion

Large tophaceous gouty deposits in the elbow may cause significant pain, local nerve compression (cubital tunnel syndrome), as well as ulceration and infection.^5^^,^^10^^,^^11^^,^^21^^,^^22^ There is a paucity of studies reporting outcomes after surgical treatment of gouty tophi in the elbow. In this case series, we describe in detail our surgical technique for open excision of gouty tophi in the olecranon region and report outcomes at a minimum of 1 year postoperatively in a series of 7 elbows.

All patients in this series reported preoperative pain and swelling that interfered with daily activities. Patients with larger swelling feared that chalky white deposits would ulcerate through their skin or elbow injury would result from direct pressure while resting their elbows. As a result, patients opted to pursue surgical treatment in concordance with previously described indications.^1^^,^^4^^,^^5^^,^^17^^,^^23^

Approaches to the surgical management of tophaceous gout affecting the elbow employing open excisional and arthroscopic tissue shaver techniques have been described in previous case reports and case series.^5^^,^^10^^,^^12^^,^^15^^,^^17^^,^^23^^,^^24^ Ozdemir et al. and Patel et al. have published case studies, which focus on patients with bilateral tophaceous gout and pseudogout of the elbow respectively.^15^^,^^17^ These authors concentrated on discussing clinical presentations, operative outcomes, and pathophysiology/disease characteristics, with limited commentary included regarding the details of the open surgical approaches that they used. Lee et al. have reported in detail on their arthroscopic shaving technique for treating tophaceous swellings using a soft tissue shaver with generally good results except in 2 patients who required postoperative skin grafting for partial skin loss in their initial series.^11^ Additionally, patients in their 3 published series required extensive postoperative wound management and inpatient stay (mean length of hospitalization >1 week across all studies).^10^, ^11^, ^12^ Straub et al. reported good results in 21 patients with open excision of gouty tophi in upper extremity but had limited follow-up of their cohort.^23^ Kumar et al. reported on 45 patients who underwent surgery for gouty tophi, describing both the indications for surgery and outcomes. Ultimately they found that wound complications are common if the indication for surgery is sepsis control.^7^ The results of our study differ fundamentally from these previous studies because the series presented here consists exclusively of gouty tophi in the elbow and provides adequate data to appropriately capture the postoperative courses of the patients in question. All patients underwent successful procedures as indicated by the absence of intraoperative and postoperative complications and the lack of recurrence of elbow tophaceous deposits at a minimum 1-year follow-up.

Wound complications continue to be a major concern after the open excision of gouty tophi. Although arthroscopic shaving technique exists as an alternative to open surgical management, we believe that there are certain important aspects of the surgical technique outlined in this study that can minimize the risk of this complication with the open approach. First, a liberal incision length should be used for excision (Fig. 3). Second, in large swellings (>5 cm), an ellipse of the skin overlying the central part of swelling should be excised along with the tophaceous material (Fig. 2). Third, the subcutaneous flaps should be kept as thick as feasible, even if it means leaving small satellite nodules of chalky white deposits on the subcutaneous flaps. We did not notice any recurrence in our case series when these small satellite deposits were left behind. Fourth, dissection should be performed with a surgical scalpel, and the use of unipolar electrocautery should be minimized or avoided. Fifth, the dead space should be managed in large swellings (>5 cm), and management should include tacking the subcutaneous flaps to the underlying fascia with absorbable sutures to minimize postoperative hematoma formation. Last, subcuticular running closure should be avoided, and postoperative splinting aids in soft tissue healing, especially in large swellings (>5 cm).

Limitations of this case series include the small number of subjects (n = 7 elbows). However, considering the relative rarity of this condition, this is a reasonable number of patients for describing the outcomes after surgical excision. This is a single-surgeon case series, with an all-male cohort based in a focal geographical region, all of which may, therefore, limit the generalizability of the conclusions drawn. However, given the limited literature on this topic, we believe this case series is of clinical importance for elbow surgeons who are inclined to manage tophaceous gout in the olecranon region with open excision.

## Conclusion

This study demonstrates that large gouty tophaceous deposits in the elbow (olecranon) can be safely excised with a low risk of infection, wound healing complications, and recurrence.

## Disclaimers

Funding: No funding was disclosed by the authors.

Conflicts of interest: The authors, their immediate families, and any research foundation with which they are affiliated have not received any financial payments or other benefits from any commercial entity related to the subject of this article.
